# Common Adverse Events from Mixing COVID-19 Vaccine Booster in Hanoi, Vietnam

**DOI:** 10.3390/vaccines11061097

**Published:** 2023-06-13

**Authors:** Pham Van Hung, Thai Duy Nguyen, Luu Thi Ha, Phung Lam Toi, Tran Hong Tram

**Affiliations:** 1 National Institute for Control of Vaccine and Biologicals, Hanoi 100000, Vietnam; hungnicvb@gmail.com (P.V.H.); thainguyenduy@hotmail.com (T.D.N.); 2 Institute of Reproductive and Child Health/Ministry of Health Key Laboratory of Reproductive Health and Department of Epidemiology and Biostatistics, School of Public Health, Peking University Health Science Center, Beijing 100191, China; luuthiha@stu.pku.edu.cn; 3 Health Strategy and Policy Institute, Ministry of Health, Hanoi 100000, Vietnam; phunglamtoi@hspi.org.vn

**Keywords:** COVID-19, vaccine, adverse events, mixing, matching, Vietnam

## Abstract

Background: Mixing vaccines was proposed as a solution to tackle supply chain interruptions during the crisis of the COVID-19 pandemic. This study aimed to investigate the safety of mixing COVID-19 vaccines for a booster dose in Hanoi, Vietnam. Method: A cross-sectional study was conducted via a telephone-based interview to identify the adverse events following COVID-19 vaccination among 719 participants in Hanoi, Vietnam. Results: In total, 45.76% of participants experienced at least one adverse event following two doses of the COVID-19 vaccine. Most of the adverse events were local effects with mild symptoms such as fever, headache, muscle pain, and/or pain at the site. In general, matching two doses in the same vaccines was not associated with the adverse events as compared to mixing vaccines (OR = 1.43, 96%CI: 0.93–2.2), except matching two doses of Pfizer (OR = 2.25, 95%CI: 1.33–3.82). Conclusion: The findings of this study suggest the overall safety of mixed vaccination. In light of the vaccine shortage, mixing vaccinations for COVID-19 prevention is a good solution. Further studies with larger cohorts and investigating immunity following mixing vaccines are needed to elucidate the mechanism.

## 1. Introduction

Since its appearance in late 2019, the COVID-19 pandemic, which was brought on by the new coronavirus SARS-CoV-2, has wreaked havoc on the world’s public health systems and economies. Researchers have been working nonstop to create secure and efficient vaccines to fight the epidemic. In order to stop the virus’s transmission and lessen the severity of COVID-19 infections, vaccinations have been essential for the general population [1,2] and vulnerable groups [3,4].

However, as is clear from public statistics, there have been several issues with this vaccine program. Some nations have suspended the use of a few COVID-19 vaccines due to several negative side effects. Additionally, providing populations with proper vaccine doses has proven challenging in developing nations. It was therefore believed that the ability to mix and match vaccines would facilitate immunization and lessen the impact of any supply chain delays, enabling vaccination programs to be more adaptable in the future [5,6,7]. This approach, which calls for the first and second doses of the COVID-19 mass immunization program to contain heterologous vaccines, might assist in resolving the aforementioned issues with vaccine shortages [7,8]. Mixing vaccines is also intended to provide a stronger immune response that will last longer and provide a better defense against the virus’s evolving variations [9,10].

On 8 March 2021, Vietnam launched the COVID-19 immunization program, first focusing on high-risk populations. The Vietnamese government has made investments in the national budget and has also received support from international organizations such as the COVAX Facility and other governments through vaccine diplomacy to ensure the COVID-19 vaccine supply, according to the Ministry of Health, in order to achieve the target of 90% vaccine coverage among the over-18 population in the COVID-19 vaccination campaign [11]. Many SARS-CoV-2 vaccines, including BNT162b2 (PfizerBioNTech), ChAdOx1 (AstraZeneca), mRNA-1273 (Moderna), and Ad26.COV2.S (Johnson & Johnson/Janssen), Sinopharm, and Sinovac were made accessible for emergency use as of December 2020.

Previous evidence in Vietnam suggested that the matching of the same COVID-19 vaccine for a booster is safe with the PfizerBioNTech [12] or mRNA-1273 (Moderna) [13]. Less is known about the safety of mixed doses between different vaccines in two doses of vaccination [14]. A study in Saudi Arabia highlighted the overall safety of mixing vaccines against COVID-19 infection [15], but only reported data of two vaccines: the Pfizer–BioNTech mRNA (BNT162b2) and Oxford–AstraZeneca (ChAdOx1 nCoV-19) vaccines. 

In a cohort of vaccinated individuals in Hanoi, Vietnam, we conducted this study to compare the short-term adverse events brought on by the mixed vaccination approach and the matched vaccine approach.

## 2. Materials and Methods

A cross-sectional study was conducted to collect data on adverse events following immunization after each dose administered in 2021.

### 2.1. Study Population

The study included individuals aged 18 and above who received a COVID-19 vaccination (Pfizer-BioNTech, Astra Zeneca, Sputnik V, Moderna, Vero Cell (Sinovac inactivated vaccine)) at the National Institute of Control for Vaccines and Biologicals (NICVB) in Hanoi in 2021. Participants had to meet the inclusion criteria of receiving two doses of the vaccine (either the same or a different type) at the NICVB and providing written informed consent for research participation.

The study selected all Hanoi residents who received their COVID-19 vaccination at the NICVB and agreed to participate in the research. In total, 719 individuals who received two doses of the vaccine at the NICVB in 2021 were included, with the time interval between doses following the Ministry of Health guidelines for each vaccine type.

### 2.2. Data Collection

Nurses conducted phone-based interviews to collect data on adverse events after each vaccine dose, including local pain or burning, fever, fatigue, muscle pain, and headache. Medical doctors collected data on shock within 30 min after vaccination. The data collection used the same questionnaire for both doses, which included two parts: demographic characteristics such as age, gender, and living area, and adverse events. The questionnaire underwent a pretest before official use.

### 2.3. Data Analysis

The data collected were entered and cleaned using Epidata version 3.1 software, and STATA version 16.0 software was utilized for the analysis. Descriptive statistics in terms of frequencies and percentages were used to describe the status of adverse events following immunization with the COVID-19 vaccine. A logistic regression analysis was conducted to identify factors influencing the rate of adverse events after immunization, including the consistency of the two vaccine doses (same or different vaccines). The independent variable used was adverse events (yes/no), with experiencing adverse events defined as having at least one symptom after immunization in either the first or second dose. The participants’ age was calculated from their date of birth to 2021, and they were categorized into three age groups: 18–34 years old, 35–54 years old, and >55 years old (reference group).

### 2.4. Ethical Consideration

The research protocol was approved by the Institutional Review Board (IRB) of the National Institute of Control for Vaccine and Biologicals (No. 0126/2022/KĐQG-HDYDD) before official data collection. All study participants provided written informed consent, and their information was kept confidential and solely used for research purposes.

## 3. Results

### 3.1. General Characteristics of Participants

Table 1 provides the demographic and clinical information of 719 vaccinated patients, including age and sex, as well as the types of COVID-19 vaccines administered for each dose and both doses. Most participants were male (59.94%) and aged between 35 and 54 years (65.09%). The most administered vaccine for the first dose was AstraZeneca (49.24%), followed by Pfizer (25.45%) and Moderna (20.45%).

For the consistency of the two doses, 35.47% of participants received both doses of AstraZeneca, 25.31% received both doses of Pfizer, and 20.31% received both doses of Moderna. In total, 14.19% of participants received two different types of vaccines for their doses (Table 1). 

### 3.2. Common Adverse Events following COVID-19 Vaccination

Table 2 illustrates the prevalence of adverse effects related to the first dose, second dose, and both doses of COVID-19 vaccines among the study population of 329 individuals. Of those participants who reported adverse effects after the first dose, 45.76% experienced at least one adverse event. The adverse event most reported by participants was pain at the vaccination site, experienced by 43.25% of those who reported adverse events. This was followed by fatigue/muscle pain at 12.10% and fever/headache, with fever and headache equally reported at 10.99% each. Shock was the least commonly reported adverse event (1.25%). The results related to the second dose and both doses were consistent with the first dose, with similar proportions of adverse effects reported. However, shock was not reported in any of the cases related to the second dose. Pain at the vaccination site was the most frequently reported adverse event, accounting for over 40% of cases, followed by fatigue/muscle pain at around 12% (Table 2). 

The proportion of individuals who experienced fever and headaches was the same, at approximately 10%, across the first dose, second dose, and both doses of the vaccine. This similarity may be because individuals who experience fever commonly also experience headaches. Additionally, the proportions of each side effect were similar between the first dose and second dose, indicating that if an individual experienced a certain side effect after the first dose, they were likely to experience the same side effect again after the second dose (Table 2).

### 3.3. Associations with Adverse Events following COVID-19 Vaccination

Table 3 presents the distribution of gender across different age groups and COVID-19 vaccine types in the study population of 682 individuals. Our analysis revealed that older age groups had a higher proportion of males (18–34 years: 50.41%; 35–54 years: 59.38%; ≥55 years: 72.22%), while younger age groups had a higher percentage of females (18–34 years: 49.59%; 35–54 years: 40.62%; ≥55 years: 27.78%). In terms of vaccine type, for the first dose, AstraZeneca was the most administered vaccine in the three age groups, followed by Pfizer and Moderna. For the second dose, Pfizer was the most frequently used vaccine in both the youngest and oldest age groups, whereas AstraZeneca was only the most frequently used vaccine in the middle-aged group (35–54 years). The findings show that Moderna recipients in the 18–34 years age group had higher odds of experiencing adverse reactions in the second dose than those who received Pfizer (OR = 3.14). Conversely, in the 35–54 years age group and the first dose, Moderna recipients had lower odds of experiencing adverse effects than those who received Pfizer (OR = 0.66). Additionally, individuals who received AstraZeneca in the 35–54 years age group of the first dose and in the 18–34 years age group of the second dose were more likely to experience adverse effects than those who received Pfizer (OR = 1.28 and OR = 4.4, respectively). The *p*-values being less than 0.001 indicate a statistically significant difference rather than a chance occurrence (Table 3). 

Table 4 presents the results of this study which examined various factors associated with adverse effects after COVID-19 vaccination in a sample of 719 individuals. The study found that the type of vaccine used and vaccine consistency across doses were significant predictors of adverse effects, while age and gender were not.

Specifically, the use of Pfizer and Moderna vaccines in the first dose was associated with higher odds of adverse effects compared with AstraZeneca (*p* < 0.0001: Moderna OR = 1.59, Pfizer OR = 2.2). This was also the case with the second dose (*p* < 0.01: Moderna OR = 1.49, Pfizer OR = 1.66). Individuals who received two doses of different vaccine types were less likely to experience adverse effects than those who received two doses of the same vaccine type. The risk of adverse effects was 2.25 times higher in individuals who received two doses of Pfizer compared to those who received two doses of different vaccine types. The risk of adverse effects with two doses of Moderna or AstraZeneca was also higher compared to those who received two doses of different vaccine types, but the odds ratios were smaller than that of Pfizer (OR = 1.66 and 1.06, respectively, for Moderna and Astra). The analysis of vaccine consistency yielded similar results, suggesting that individuals who received two doses of different vaccine types had a lower likelihood of adverse effects than those who received two doses of the same vaccine type (OR = 1.43, *p* = 0.1) (Table 4). 

The results indicate that the type of vaccine used in the first dose may have a significant impact on the occurrence of adverse effects, with certain vaccine types being associated with a higher risk. Individuals who received the Pfizer vaccine as their initial dose had a higher risk of experiencing adverse effects compared to those who received the AstraZeneca vaccine. The odds ratios for different adverse effects ranged from 1.74 to 2.17. Further analyses revealed that individuals who received two doses of Pfizer were also more likely to experience adverse effects compared to those who received two doses of different vaccine types. The odds ratios for adverse effects associated with two doses of Pfizer ranged from 2.22 to 3.41, depending on the type of adverse effect. Similar associations were observed for individuals who received two doses of AstraZeneca or Moderna, and the association observed in the first dose (*p* < 0.0001) was stronger than that observed in the second dose (*p* = 0.005). It is noteworthy that age and gender were not significant predictors of adverse effects, and the association between vaccine consistency across doses and adverse effects was weak (*p* = 0.125) (Table 5). 

Table 6 provides information regarding the association between various risk factors and the incidence of shock following COVID-19 vaccination. Our analysis indicated that there was a weak association between the type of vaccine used and the occurrence of shock (*p* = 0.142). Specifically, individuals who received the Pfizer vaccine had greater odds of experiencing shock compared to those who received AstraZeneca (OR = 3.29), whereas individuals who received the Moderna vaccine had lower odds of experiencing shock compared to those who received AstraZeneca (OR = 0.8). Additionally, we found no significant association between gender or age group and the risk of shock after vaccination. Furthermore, it is noteworthy that all cases of shock occurred after the first dose, and no instances of shock were observed after the second dose (Table 6). 

## 4. Discussion

Many countries have allowed the use of two separate vaccinations for the prime and boost doses due to the huge demand for COVID-19 vaccines on a global scale. However, there is a lack of knowledge on the effectiveness and, more crucially, the safety of this mixed vaccination strategy. Since September 2021, the Vietnamese government has permitted the mixing of the COVID-19 vaccine for the second booster. Hence, this study was designed to assess the side effects associated with this mixed vaccination approach.

To the best of our knowledge, the present study is the first study in Vietnam that provides safety data on various combined vaccination strategies for COVID-19 prevention. While previous studies in Vietnam considered the safety of two matching doses of Pfizer [12], Moderna [13] vaccines, or a single dose [16], this study delves deeper into the safety implications of combining different types of vaccines for the second booster dose. The study confirmed previous evidence on the safety of mixed COVID-19 vaccines [10]. The data showed that only 45.76% of participants experienced at least one adverse event after receiving two doses of the COVID-19 vaccine (Table 1). Most of the adverse events were local effects with mild symptoms such as fever, headache, muscle pain, and/or pain at the site. Only nine participants (1.25%) experienced symptoms of shock following the first dose of the vaccine, and all were managed well without any mortality. No correlations were found between shock symptoms and participants’ age, gender, or the type of vaccine received (Table 6). 

The present study found no significant increase in side effects with the mixed vaccination approach compared to the matched vaccination method. There was no statistically significant association found between matching two doses of the same vaccine and adverse events (OR = 1.43, 96%CI: 0.93–2.2) (Table 4). This finding aligns with previous evidence attesting to the safety of mixing and matching COVID-19 vaccines. A Spanish study reported that 448 individuals received the Oxford AstraZeneca vaccine as their first dose and the Pfizer BioNTech vaccine as their second dose. These individuals experienced minor side effects, and blood tests revealed a robust antibody response two weeks after the second dose. Trials involving a mix-and-match approach have not reported any severe side effects to date [9]. Similar results were obtained by Charité, Saarland, and CombiVacS; these vaccinations’ side effects were comparable to those of two doses of the same vaccine [6]. However, the ComCOV research shows that administering two doses of mixed vaccination rather than combining two doses of the same vaccine might result in additional adverse effects [8]. This was also in accordance with the finding of a study in Saudi Arabia, which found that compared to the matched vaccination technique, the mixed vaccination approach was associated with a higher rate of side effects [15].

Notably, this study’s finding suggested that a matched vaccine tended to associate with higher adverse events in matching two doses of Pfizer (OR = 2.25, 95%CI: 1.33–3.82). Meanwhile, there was no statistical significance in the association of adverse events when matching two doses of AstraZeneca, Moderna, and other vaccines as compared to mixed vaccines (Table 4). The matching two doses of Pfizer were associated with a higher rate of fever (OR = 3.41, 95%CI: 1.24–11.69), headache (OR = 3.41, 95%CI: 1.24–11.69), fatigue/muscle pain (OR = 2.59, 95%CI: 1.06–7.26), and pain at site (OR = 2.22, 95%CI: 1.31–3.82) as compared to the mixed vaccines. Meanwhile, the matching of two doses of Moderna was only statistically associated with a higher rate of fever (OR = 3.15, 95%CI: 1.1–11.07), headache (OR = 3.15, 95%CI: 1.1–11.07), and pain at the site (OR = 1.82, 95%CI: 1.05–3.2) as compared to the mixed vaccines. There was no statistical significance associated between matching two doses of AstraZeneca with the aforementioned adverse events (Table 5). 

In a study in Vietnam, Xuan et al. [12] reported that the percentage of adverse events following the two doses of the Pfizer vaccine was 18.5%. In our study, the percentage of adverse events after two doses of the Pfizer vaccine was higher, with 32.22% of participants having at least one adverse event (Table 4). This could be explained by the characteristics of participants in the vaccination campaign. Even though our study did not collect data regarding the occupation of participants, in general, the participants receiving vaccination in the NICVB during the time of this study were mainly lay people. Meanwhile, in the Xuan et al. study [12], a substantial proportion of participants were businessmen and officers (white collar), who might have better health conditions. 

In this study, the adverse events following COVID-19 vaccination were not associated with gender and age. Our finding is similar to a study in Saudi Arabia that showed that gender was not associated with adverse events following COVID-19 vaccination [15]. Conversely, another study showed that age ≤55 years and females were more likely to have adverse events after COVID-19 vaccination [12]. Other studies also reported that females had higher odds of experiencing adverse events compared to males [17,18]. Further studies with larger cohorts are needed to clarify the relationship between age and gender with adverse events following the matching and mixing of vaccines. 

In this study, there were only nine cases of experiencing shock following the first doses of vaccination. The results showed that there was no statistical association between having shock events and the type of vaccines. This was in light of previous evidence that mentioned the rare cases of shock after being vaccinated [19], and there was no difference between the type of vaccines (Pfizer/BioNTech or Moderna) [20]. 

This study has some limitations. First, as is the nature of the cross-sectional study, any causal-effect relationship regarding the common adverse events following COVID-19 vaccination could not be drawn. Additionally, the study could not evaluate the effectiveness of mixing vaccine strategies in the prevention of COVID-19. Second, we did not investigate the duration or average period of how long the symptom lasted. Third, the study population receiving the COVID-19 vaccine was not representative of the whole population in Vietnam, especially those living in rural areas. Furthermore, the occupation of participants was not collected in our study. Fourth, we could not separate the participants who were already infected and diagnosed with COVID-19 before receiving the vaccine, as the immunity of the recovered patients can react differently to the vaccine compared to those naïve with the disease [21]. Further studies might be interested in comparing the adverse events following COVID-19 vaccination between these two groups to draw more precise conclusions. Finally, we did not collect data on the participants’ medical history such as allergic disease or chronic disease. However, previous studies in Vietnam showed that these factors did not associate with adverse events following vaccination. 

## 5. Conclusions

The findings of this study suggest the overall safety of mixed vaccination. In light of the vaccine shortage, the mixing of vaccinations for COVID-19 prevention is a good solution. Further studies with larger cohorts and investigating the immunity following mixing vaccines are needed to elucidate the mechanism. 

## Figures and Tables

**Table 1:** Demographic characteristics of participants (n = 719).

Variables		n	Percentage %
Age group	Total	719	100%
	18–34 years old	130	18.08%
	35–54 years old	468	65.09%
	55–73 years old	121	16.83%
Gender	Total	719	100%
	Male	431	59.94%
	Female	288	40.06%
Dose 1 by vaccines	Total	719	100%
	Astra	354	49.24%
	Moderna	147	20.45%
	Pfizer	183	25.45%
	Others	35	4.87%
Dose 2 by vaccines	Total	719	100%
	Astra	256	35.61%
	Moderna	150	20.86%
	Pfizer	277	38.53%
	Others	36	5.01%
Two doses by vaccines	Total	719	100%
	Two doses are not the same	102	14.19%
	Two doses are Astra	255	35.47%
	Two doses are Moderna	146	20.31%
	Two doses are Pfizer	182	25.31%
	Two doses are Others (same vaccines)	34	4.73%
Consistency of two doses	Total	719	100%
	Two doses are different vaccines	102	14.19%
	Two doses are the same vaccines	617	85.81%
Adverse events	Total	719	100%
	Yes	329	45.76%
	No	390	54.24%

**Table 2:** Distribution of adverse effects between two doses and both doses (n = 329).

Adverse Effects		n (%)
The first dose	No adverse effects	390 (54.24%)
	At least one adverse effect	329 (45.76%)
	Shock	9 (1.25%)
	Fever	79 (10.99%)
	Headache	79 (10.99%)
	Fatigue/muscle pain	87 (12.10%)
	Pain at the site of the vaccination	311(43.25%)
The second dose	No adverse effects	390 (54.24%)
	At least one adverse effect	329 (45.76%)
	Shock	0 (0%)
	Fever	79 (10.99%)
	Headache	79 (10.99%)
	Fatigue/muscle pain	87 (12.10%)
	Pain at the site of the vaccine	311(43.25%)
Both doses	No adverse effects	390 (54.24%)
	At least one adverse effect	329 (45.76%)
	Shock	9 (1.25%)
	Fever	79 (10.99%)
	Headache	79 (10.99%)
	Fatigue/muscle pain	87 (12.10%)
	Paint at the site of the vaccine	311(43.25%)

**Table 3:** Distribution of gender based on age group and different types of vaccines (n = 682).

**(A) The First Dose**						
**Age Group**	**Vaccine (n)**	**n (%)**	**Gender for Adverse Effects**		**Odds Ratio**	***p*-Value**
			**Male** **(n = 408)**	**Female** **(n = 274)**		
18–34 years old	Total	121	61	60		
		(100%)	(50.41%)	(49.59%)		
	Astra	57	32	25	2.67	0.064
		(100%)	(56.14%)	(43.86%)	(1.07–6.72)	
	Moderna	33	19	14	2.85	
		(100%)	(57.58%)	(42.42%)	(1.03–7.92)	
	Pfizer	31	10	21	1	
		(100%)	(32.26%)	(67.74%)		
35–54 years old	Total	453	269	184		
		(100%)	(59.38%)	(40.62%)		
	Astra	255	163	92	1.28	0.028 *
		(100%)	(63.92%)	(36.08%)	(0.81–2.02)	
	Moderna	86	41	45	0.66	
		(100%)	(47,67%)	(52.33%)	(0.37–1.16)	
	Pfizer	112	65	47	1	
		(100%)	(58.04%)	(41.96%)		
≥55 years old	Total	108	78	30		
		(100%)	(72.22%)	(27.78%)		
	Astra	41	30	11	1.07	0.985
		(100%)	(73.17%)	(26.83%)	(0.40–2.86)	
	Moderna	28	20	8	0.98	
		(100%)	(71.43%)	(28.57%)	(0.33–2.88)	
	Pfizer	39	28	11	1	
		(100%)	(71.79%)	(28.21)		
**(B) The second dose**						
**Age Group**	**Vaccine (n)**	**n (%)**	**Gender for Adverse Effects**		**Odds Ratio**	***p*** **-Value**
			**Male** **(n = 408)**	**Female** **(n = 274)**		
18–34 years old	Total	121	61	60		
		(100%)	(50.41%)	(49.59%)		
	Astra	39	26	13	4.4	0.002 *
		(100%)	(66.67%)	(33.33%)	(1.78–10.86)	
	Moderna	34	20	14	3.14	
		(100%)	(58.82%)	(41.18%)	(1.26–7.85)	
	Pfizer	48	15	33	1	
		(100%)	(31.25%)	(68.75%)		
35–54 years old	Total	453	269	184		
		(100%)	(59.38%)	(40.62%)		
	Astra	189	119	70	1.09	0.059
		(100%)	(62.96%)	(37.04%)	(0.71–1.66)	
	Moderna	87	42	45	0.60	
		(100%)	(48.28%)	(51.72%)	(0.36–1.00)	
	Pfizer	177	108	69	1	
		(100%)	(61.02%)	(38.98%)		
≥55 years old	Total	108	78	30		
		(100%)	(72.22%)	(27.78%)		
	Astra	28	20	8	1.04	0.875
		(100%)	(71.43%)	(28.57%)	(0.38–2.88)	
	Moderna	29	22	7	1.31	
		(100%)	(75.86%)	(24.14%)	(0.46–3.71)	
	Pfizer	51	36	15	1	
		(100%)	(70.59%)	(29.41%)		

(*): *p* < 0.05.

**Table 4:** Association of factors with the adverse effects after receiving COVID-19 vaccines (n = 719).

Factors		Total n (%)	Adverse Effects		Odds Ratio OR (95%CI)	*p*-Value
			Yes n (%)	No n (%)		
Age group	Both doses	719	329	390		0.623
		(100%)	(100%)	(100%)		
	18–34 years old	130	57	73	1	
		(18.08%)	(17.33%)	(18.72%)		
	35–54 years old	468	212	256	1.06	
		(65.09%)	(64.44%)	(65.64%)	(0.7–1.6)	
	55–73 years old	121	60	61	1.26	
		(16.83%)	(18.24%)	(15.64%)	(0.74–2.13)	
	Dose 1	719	329	390		0.623
		(100%)	(100%	(100%)		
	18–34 years old	130	57	73	1	
		(18.08%)	(17.33%)	(18.72%)		
	35–54 years old	468	212	256	1.06	
		(65.09%)	(64.44%)	(65.64%)	(0.7–1.6)	
	55–73 years old	121	60	61	1.26	
		(16.83%)	(18.24%)	(15.64%)	(0.74–2.13)	
	Dose 2	719	327	392		0.597
		(100%)	(100%)	(100%)		
	18–34 years old	130	57	73	1	
		(18.08%)	(17.43%)	(18.62%)		
	35–54 years old	468	210	258	1.04	
		(65.09%)	(64.22%)	(65.82%)	(0.69–1.57)	
	55–73 years old	121	60	61	1.26	
		(16.83%)	(18.35%)	(15.56%)	(0.74–2.13)	
Gender	Both doses	719	329	390		0.905
		(100%)	(100%)	(100%)		
	Male	431	198	233	1	
		(59.94%)	(60.18%)	(59.74%)		
	Female	288	131	157	0.98	
		(40.06%)	(39.82%)	(40.26%)	(0.73–1.32)	
	Dose 1	719	329	390		0.905
		(100%)	(100%)	(100%)		
	Male	431	198	233	1	
		(59.94%)	(60.18%)	(59.74%)		
	Female	288	131	157	0.98	
		(40.06%)	(39.82%)	(40.26)	(0.73–1.32)	
	Dose 2	719	327	392		0.881
		(100%)	(100%)	(100%)		
	Male	431	197	234	1	
		(59.94%)	(60.24%)	(59.69%)		
	Female	288	130	158	0.98	
		(40.06%)	(39.76%)	(40.31%)		
Dose 1 by vaccines	Astra	354	138	216	1	<0.0001 *
		(49.24%)	(41.95%)	(55.38%)		
	Moderna	147	74	73	1.59	
		(20.45%)	(22.49%)	(18.72%)	(1.06–2.38)	
	Pfizer	183	107	76	2.2	
		(25.45%)	(32.52%)	(19.49%)	(1.51–3.22)	
	Others	35	10	25	0.63	
		(4.87%)	(3.04%)	(6.41%)	(0.26–1.4)	
Dose 2 by vaccines	Astra	256	101	155	1	0.003 *
		(35.61%)	(30.70%)	(39.74%)		
	Moderna	150	74	76	1.49	
		(20.86%)	(22.49%)	(19.49%)	(0.97–2.29)	
	Pfizer	277	144	133	1.66	
		(38.53%)	(43.77%)	(34.10%)	(1.16–2.38)	
	Others	36	10	26	0.59	
		(5.01%)	(3.04%)	(6.67%)	(0.24–1.33)	
Two doses by vaccines	Two doses are not the same	102	39	63	1	<0.0001 *
		(14.19%)	(11.85%)	(16.15%)		
	Two doses are Astra	255	101	154	1.06	
		(35.47%)	(30.70%)	(39.49%)	(0.65–1.75)	
	Two doses are Moderna	146	74	72	1.66	
		(20.31%)	(22.49%)	(18.46%)	(0.96–2.87)	
	Two doses are Pfizer	182	106	76	2.25	
		(25.31%)	(32.22%)	(19.49%)	(1.33–3.82)	
	Two doses are Others (same vaccines)	34	9	25	0.58	
		(4.73%)	(2.74%)	(6.41%)	(0.22–1.46)	
Consistency of two doses	Two doses are different vaccines	617	290	327	1	0.1
		(100%)	(88.15%)	(83.85%)		
	Two doses are the same vaccines	102	39	63	1.43	
		(14.19%)	(11.85%)	(16.15%)	(0.93–2.2)	

(*) *p* < 0.01.

**Table 5:** Association of the risk factors with different type of common adverse effects after receiving vaccines.

Factors		Type of Adverse Effects							
		Fever		Headache		Fatigue/ Muscle Pain		Pain at Site	
		Odds Ratio	*p*-Value	Odds Ratio	*p*-Value	Odds Ratio	*p*-Value	Odds Ratio	*p*-Value
Age group	Both doses		0.598		0.598		0.925		0.402
	18–34 years old	1		1		1		1	
	35–54 years old	0.81 (0.44–1.56)		0.81 (0.44–1.56)		0.9 (0.49–1.73)		1.03 (0.68–1.56)	
	55–73 years old	0.67 (0.27–1.59)		0.67 (0.27–1.59)		0.87 (0.38–1.98)		1.34 (0.79–2.27)	
	Dose 1		0.598		0.598		0.925		0.402
	18–34 years old	1		1		1		1	
	35–54 years old	0.81 (0.44–1.56)		0.81 (0.44–1.56)		0.9 (0.49–1.73)		1.03 (0.68–1.56)	
	55–73 years old	0.67 (0.27–1.59)		0.67 (0.27–1.59)		0.87 (0.38–1.98)		1.34 (0.79–2.27)	
	Dose 2		0.598		0.598		0.925		0.402
	18–34 years old	1		1		1		1	
	35–54 years old	0.81 (0.44–1.56)		0.81 (0.44–1.56)		0.9 (0.49–1.73)		1.03 (0.68–1.56)	
	55–73 years old	0.67 (0.27–1.59)		0.67 (0.27–1.59)		0.87 (0.38–1.98)		1.34 (0.79–2.27)	
Gender	Both doses		0.192		0.192		0.462		0.693
	Male	1		1		1		1	
	Female	1.37 (0.85–2.18)		1.37 (0.85–2.18)		1.18 (0.75–1.86)		0.94	
	Dose 1		0.192		0.192		0.462		0.693
	Male	1		1		1		1	
	Female	1.37 (0.85–2.18)		1.37 (0.85–2.18)		1.18 (0.75–1.86)		0.94	
	Dose 2		0.192		0.192		0.462		0.693
	Male	1		1		1		1	
	Female	1.37 (0.85–2.18)		1.37 (0.85–2.18)		1.18 (0.75–1.86)		0.94	
Dose 1 by vaccine	Astra	1	0.032 *	1	0.032 *	1	0.097	1	<0.0001 *
	Moderna	1.79 (0.94–3.37)		1.79 (0.94–3.37)		1.47 (0.78–2.7)		1.75 (1.17–2.64)	
	Pfizer	1.95 (1.08–3.52)		1.95 (1.08–3.52		1.74 (0.99–3.02)		2.17 (1.48–3.18)	
Dose 2 by vaccine	Astra	1	0.423	1	0.423	1	0.676	1	0.005 *
	Moderna	1.5 (0.77–2.92)		1.5 (0.77–2.92)		1.27		1.69 (1.1–2.6)	
	Pfizer	1.25 (0.7–2.27)		1.25 (0.7–2.27)		1.21		1.71 (1.19–2.45)	
Two doses by vaccines	Two doses are not the same	1	0.035 *	1	0.035 *	1	0.115	1	<0.0001 *
	Two doses are Astra	2.04 (0.74–7.03)		2.04 (0.74–7.03)		1.68 (0.69–4.72)		1.03 (0.62–1.73)	
	Two doses are Moderna	3.15 (1.1–11.07)		3.15 (1.1–11.07)		2.2 (0.86–6.4)		1.82 (1.05–3.2)	
	Two doses are Pfizer	3.41 (1.24–11.69)		3.41 (1.24–11.69)		2.59 (1.06–7.26)		2.22 (1.31–3.82)	
Consistency of two doses	Two doses are different vaccines	1	0.034 *	1	0.034 *	1	0.08	1	0.125
	Two doses are the same vaccines	2.64 (1.04–6.71)		2.64 (1.04–6.71)		2.02 (0.91–4.51)		1.4 (0.91–2.17)	

(*) *p* < 0.05.

**Table 6:** Association of the risk factors with shock after receiving vaccines.

Factors		Total n (%)	Shock (*)		Odds Ratio	*p*-Value
			Yes	No		
Age group	Total	719 (100%)	9 (1.25%)	710 (98.75%)		1
	18–34 years old	130 (100%)	1 (0.77%)	129 (99.23%)	1	
	35–54 years old	468 (100%)	7 (1.50%)	461 (98.50%)	1.96 (0.25–88.93)	
	55–73 years old	121 (100%)	1 (0.83%)	120 (99.17%)	1.07 (0.01–84.98)	
Gender	Total	719 (100%)	9 (1.25%)	710 (98.75%)		0.747
	Male	431 (100%)	6 (1.39%)	425 (98.61%)	1	
	Female	288 (100%)	3 (1.04%)	285 (98.96%)	0.75 (0.18–3.01)	
Type of vaccines	Total	682 (100%)	9 (1.32%)	673 (98.68%)		0.142
	Astra	353 (100%)	3 (0.85%)	350 (99.15%)	**1**	
	Moderna	147 (100%)	1 (0.68%)	146 (99.32%)	0.8 (0.02–10.05)	
	Pfizer	182 (100%)	5 (2.75%)	177 (97.25%)	3.29 (0.63–21.41)	

(*) All shocked cases occur in the first dose of vaccination. This table’s results are based on Fisher’s exact test (2-sided).

## Data Availability

All the data available are provided in this paper.
